# Using a community based survey of healthcare seeking behavior to estimate the actual magnitude of influenza among adults in Beijing during 2013-2014 season

**DOI:** 10.1186/s12879-017-2217-z

**Published:** 2017-02-03

**Authors:** Xiaoli Wang, Shuangsheng Wu, Peng Yang, Hongjun Li, Yanhui Chu, Yaqing Tang, Weiyu Hua, Haiyan Zhang, Chao Li, Quanyi Wang

**Affiliations:** 1 Beijing Center for Disease Prevention and Control, 16 Hepingli Middle Street, Beijing, 100013 China; 2 Tongzhou District Center for Disease Prevention and Control, No.1 Luhe Middle School North Street, Tongzhou District, Beijing, 101100 China; 3 Xicheng District Center for Disease Prevention and Control, No.38 Dewai Avenue, Xicheng District, Beijing, 100120 China; 4 Changping District Center for Disease Prevention and Control, No.7 Gulou North Street, Changping District, Beijing, 102200 China; 5 Haidian District Center for Disease Prevention and Control, No. 5 Xibeiwang Second Street, Haidian District, Beijing, 100094 China; 6 Dongcheng District Center for Disease Prevention and Control, No. 5 Bingmasi North Lane, Beijing, 10009 China; 7 Huairou District Center for Disease Prevention and Control, No.23 Fule North, Huairou District, Beijing, 101400 China

**Keywords:** Burden, Influenza, Healthcare seeking behavior, Consultation rate

## Abstract

**Background:**

Due to a lack of survey of health care seeking behavior for influenza, the actual magnitude of influenza in Beijing of China has not been well described.

**Methods:**

During 2013–2014 influenza season, two cross-sectional household surveys were carried out respectively during the epidemic and non-epidemic season of influenza. A structured survey was undertaken with individuals who were ≥18 years selected by a multistage random sampling method in the study. Health care seeking behaviors were then examined to estimate the actual case number of influenza, using a multiplier model.

**Results:**

A total of 14,665 adults were interviewed. 61.9% of ILI cases consulted a physician. The consultation rate during epidemic period is higher than that during non-epidemic period (67.9% vs. 52.3%). Similarly, the proportion of healthcare usage of general hospital during epidemic period is higher than that was during non-epidemic period (27.1% vs. 19.0%, *p* = 0.008). Lack of insurance and education reduced healthcare seeking significantly in this study. It was estimated that there were 379,767 (90% CI = [281,934, 526,565]) confirmed cases of influenza amongst adults in Beijing, during 2013–2014 influenza season, with an incidence rate of 2.0%.

**Conclusions:**

The surveillance system for ILI and virological data has the potential to provide baseline case number to estimate the actual annual magnitude of influenza. Given the changes in healthcare seeking behavior over time, sentinel surveillance on healthcare seeking behavior are required to be established for better estimate of the true case number of influenza.

## Background

Influenza, one of the most common infectious diseases, is a highly contagious airborne disease, occurs all over the world, with an annual global attack rate estimated at 5–10% in adults and 20–30% in children. The World Health Organization estimates that worldwide, annual influenza epidemics result in about 3–5 million cases of severe illness and about 250,000 to 500,000 deaths [1]. However, there were only 10,209 cases of influenza reported in Beijing of China during 2014, with an attack rate estimated at 0.04% in adults and 0.36% in children (<5 years), which was much lower than the world average level. Since not all of influenza cases would consult a physician and influenza is confirmed virologically only in a small fraction of cases, the actual magnitude of influenza has not been well established to date in Beijing, China. The proportion of cases seeking medical attention for influenza is required for estimating the case number of influenza. Some studies have described the healthcare seeking behavior for influenza [2–4]. However, few studies have been conducted to examine the consultation rate of influenza in China. Due to uncertainties in generalizing these results to Beijing of China, located in the temperate zone of the Northern Hemisphere, which is one of the most populous cities in the world and a major hub for the national transportation networks. Additionally, the differences in the healthcare system and the socio-cultural differences in healthcare usage may contribute to the differences in the consultation rate between different regions [5]. Moreover, this rate can vary with the change in the virological activity overtime. Therefore, a community based survey was conducted respectively during the epidemic and non-epidemic season of influenza to assess the healthcare usage systematically. Results from this survey were then used to estimate the true number of symptomatic case of influenza in Beijing during the influenza season 2013–2014. This activity will enable a better understanding the health care seeking behavior and consequently the actual incidence of influenza, which will be helpful for the development of effective influenza prevention strategies as well as pandemic influenza control measures.

## Methods

### Survey of healthcare seeking behaviour

Influenza occurs all year round in Beijing, from week 27 through week 26 of the next year. Epidemic and non-epidemic period of influenza are defined according to the activity of influenza virus. In general, annual epidemic period of influenza usually starts in week 40 (around October) and ends in week 13 of the next year (around March). The remaining weeks when the positive rate of influenza virus is usually low are defined as non-epidemic period (week 27 to week 39 and week 14 to week 26 of the next year). Considering the fact that healthcare seeking behavior of cases can vary with the change in the virological activity of influenza overtime. We carried out two retrospective cross-sectional household surveys respectively in epidemic period (from December 6^th^, 2013 through January 14^th^, 2014) and non-epidemic period (from May 21st through July 4th, 2014). Participants were residents who (1) were older than 18 years and had continuously lived in Beijing for more than half a year, (2) were able to communicate with others and willing to give their informed consent to participate. Potential participants were screened for eligibility at the beginning of survey. Residents were excluded if they were foreigners, or were unable to communicate in Mandarin. Multi-stage sampling technique was adopted on the basis of the sixth national census data in 2010 in Beijing, China. Beijing is administratively divided into 16 districts. These districts can further be divided into urban and suburban districts based on population density. At the first stage, a sample of primary sampling units (districts) was drawn. Then, stratified sampling was further employed to recruit subjects by age (18~,50~,and >60) and gender. A total of twelve subgroups were identified. At least 600 subjects were recruited from each subgroup. In this study, three suburban districts and three urban districts were firstly randomly selected from 16 districts. At the next stage, five sub-districts were randomly selected from each selected district. Finally, a total of 30 sub-districts were confirmed as survey locations. The sampling quotas of each sub-district were proportional to their population size. Finally, in each sub-district, proportional stratified sampling was used to select subjects based on the age and sex distribution of residents living in each selected sub-district. All participants provided verbal consent before the survey and were asked to complete the questionnaire by themselves or with the help of trained study staff if they had difficulty with reading or writing. A pilot study was conducted before December 2013 to refine the questionnaire. The questionnaire consisted of three sections: demographics (gender, age, educational level, and residential district name); history of influenza-like illness; and related health care seeking behavior. To be specific, participants were asked whether they had previously had any episodes of ILI (self-defined) within specified duration before being interviewed. To mitigate the recall bias, we asked participants if they had any episode of ILI within a short duration. During influenza epidemic, participants were asked if any episode of ILI occurred within two weeks. However, during non-epidemic period when the incidence of ILI decreased to baseline level, we expanded the duration into three months.

ILI case was defined by participants themselves or with the help of trained study staff if they had difficulty in the diagnosis of ILI. In this study, ILI is defined according the WHO guidelines, which included, fever (≥ 38.0 °C) and cough or sore throat, within a related period before the date of interview. Ethical approval was granted by the Ethics Committee and Beijing center for disease prevention and control.

### Disease burden estimation

**Fig. 1:**
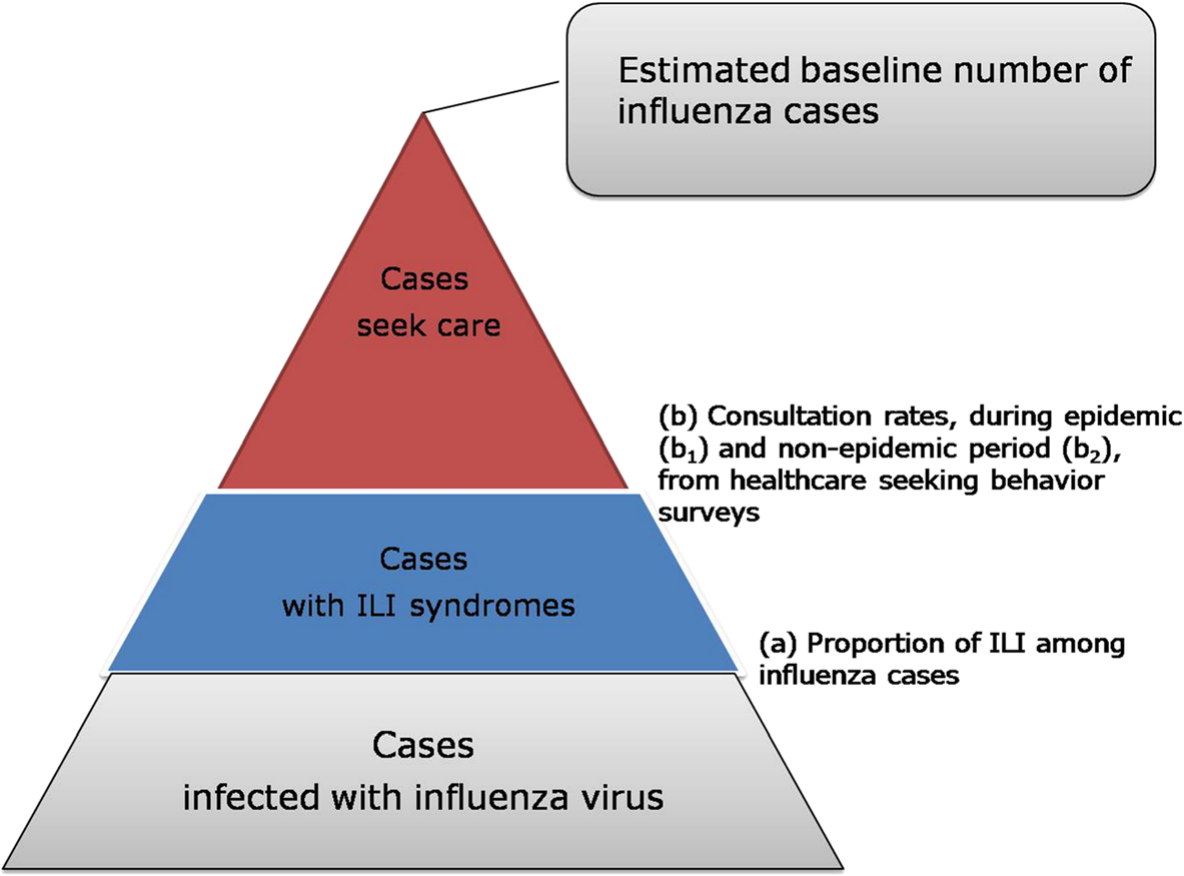
Schematic figure of multiplier model for estimates of the actual magnitude of influenza during 2013–2014 season in Beijing^#^ (^#^ At the top of the pyramid is the estimated baseline number of influenza case. It was equal to the sum of the product of weekly ILI consultations aged ≥ 18 and weekly positive isolation rate of influenza virus)

In this study, multiplier model was used to estimate the burden of influenza in Beijing, stratified by age. As shown in Fig. 1, at the bottom of the pyramid is the actual number of cases infected with influenza virus. However, only a fraction of cases infected have ILI syndromes and not all of those influenza cases with ILI syndromes seek health care. Thus at the top of the pyramid- the baseline number of influenza case that we can estimate from our surveillance data represents only a small fraction of the actual magnitude of influenza. In this study, the estimated number of total influenza cases was calculated by multiplying the estimated baseline number by multiplier.

The baseline number of influenza case equaled to the sum of the product of the weekly ILI case number (*N*_*w*_) in secondary and tertiary hospitals (levels 2, 3) and the corresponding weekly positive rate (*P*_*w*_) of influenza among case-patients with ILIs. The multiplier was the reciprocal of the proportion of ILI among influenza cases (*a*) and consultation rate (*b*). Due to the change in healthcare usage over the course of the influenza season, we used two consultation rates for this model: consultation rate during epidemic period (*b*_*1*_) and consultation rate during non-epidemic period (*b*_*2*_). The baseline number of influenza case was consequently divided into two parts: *B*_*1*_ and *B*_*2*_, (see eq. 1).

1 $$ \mathrm{Actual}\ \mathrm{number}\ \mathrm{of}\ \mathrm{influenza}\ \mathrm{cases}\kern0.5em =\kern0.5em \frac{B_1}{a{ b}_1}+\frac{B_2}{a{ b}_2}=\frac{{\displaystyle \sum_{w1}{P}_{w1}{N}_{w1}}}{a{ b}_1}+\frac{{\displaystyle \sum_{w2}{P}_{w2}{N}_{w2}}}{a{ b}_2} $$

Here, *B*_*1*_ and *B*_*2*_ were respectively the number of total baseline number estimated during epidemic period and non-epidemic period. *N*_*w1*_ *and N*_*w2*_ were the weekly ILI case number during epidemic and non-epidemic period. *P*_*w1*_ *and P*_*w2*_ were respectively the weekly positive rate of influenza among case-patients with ILIs during two periods. *w*_*1*_ was the weeks during the epidemic period of influenza, and *w*_*2*_ was the weeks during the non-epidemic period.

Accounting for variability and uncertainty in a and b, we used a multiplier model (Impact2009, version 1.0) developed by United States Centers for Disease Control and Prevention (CDC) to estimate the median, and 90% CIs for the actual number of influenza cases. For each parameter (*a* and *b*) included in the model, we used uniform probability distributions that covered a range of minimum to maximum values, from which the model randomly sampled 1,000 iterations [6].

Hospitals in Beijing are classified into 3 levels, depending on their size and the techniques, equipment, and staff available [7]. Hospitals at level 3 had the highest level of healthcare services. Based on this, hospitals are designated as primary, secondary and tertiary institutions. Consultation rate was defined as the proportion of individuals with self-defined ILI that sought healthcare in level 2/3 hospitals, which can be obtained from the survey of healthcare seeking behavior above. Proportion of ILI among influenza cases can be obtained from literatures previously published. From literatures previously published, we applied a parameter value of 30–70% to proportion of ILI among influenza cases [8–11]. Weekly ILI case number and weekly positive rate of influenza by subtype were extracted from influenza surveillance system, which included ILI surveillance and virological surveillance. ILI surveillance in Beijing was launched in 2007. Daily number of ILI cases was recorded in this surveillance system. Pharyngeal swab specimens from the ILIs case-patients (within 3 days of symptom onset from patients who had not received antiviral drugs) were randomly collected by designated staff. The specimens were tested by collaborating laboratories and the weekly positive rate of influenza by subtype was recorded [12].

### Data analysis

95% confidence intervals (CIs) for self-defined influenza cases seeking healthcare, stratified by age were calculated using the normal approximation or based on bootstrap percentile. Chi-square test was used to examine the independent associations between respondents’ characteristics and the healthcare usage. Multivariate logistic regression analysis was then employed to assess the effects of socio-demographic characteristics on individuals’ healthcare seeking behaviors. In order to include all potential factors which may influence individual healthcare behavior, we merged two databases during epidemic period and non-epidemic period. Starting with all candidate variables being included and then we removed non-significant variables using step-wise elimination. Confounding was evaluated by adding each excluded variable back into the final model individually. We examined whether changes in the β-coefficients of the included variables ≥10% or not. If the β-coefficients of the included variables exceeded 10%, the variable was retained in the model. Data analyses were performed using SPSS statistical software package version 20.0 (IBM SPSS Inc., Chicago, IL, USA). All statistical tests were 2-sided, and statistical significance was set at *p* value less than 0.05.

## Results

### Demographic characteristics of participants and report of ILI

In 2013–2014 influenza season, a total of 14,665 adults were interviewed. The median response rate was 70.8%. No statistically significant difference were found in the distribution by age (*p* = 0.112) and gender (*p* = 0.267). The age for all respondents ranges from 18 to 92 (median age, 45.1). 84.6% were married and 73.4% were employed. 6.3% were unemployed and 3.5% were students. Around 32.4% of the respondents are undergraduates or postgraduates, while 2.3% of them are illiterates.

As shown in Table 1, 490 (6.7%) of those subjects reported that they had an episode of influenza like illness (ILI) within 2 weeks during epidemic period. No significant differences were observed in the occurrence of self-defined ILI by age, gender, education, and status of marriage and employment.

**Table 1:** Demographic characteristic of participants and reported ILI during epidemic and non-epidemic period

Characteristics	Reported ILI within two weeks during epidemic period			Reported ILI within 3 months during non-epidemic period		
	n	% Yes	95% CI	n	% Yes	95% CI
All respondents	7320	6.7	6.1–7.3	7345	4.2	3.7–4.7
Age						
18–59	5870	6.6	6.0–7.3	5934	3.9	3.4–4.4
≥ 60	1450	7.0	5.7–8.3	1420	5.3	4.1–6.4
Gender						
Male	3647	6.1	5.4–6.9	3618	3.9	3.3–4.5
Female	3673	7.2	6.4–8.1	3736	4.4	3.8–5.1
Level of education						
Less than high school	2793	6.6	5.7–7.6	2386	3.9	3.2–4.6
High school graduate	2146	6.7	5.6–7.7	2237	4.1	3.3–4.9
College school graduate	2330	7.3	6.2–8.3	2406	4.5	3.7–5.4
Marriage status						
Married	829	7.2	5.5–9.0	937	3.1	2.0–4.2
Divorced	6115	6.6	6.0–7.2	6083	4.3	3.8–4.8
Unmarried	267	11.2	7.4–15.0	332	4.8	2.5–7.1
Employment status						
Student	250	6.8	3.7–9.9	272	4.0	1.7–6.4
Employed	5317	6.5	5.8–7.2	5430	4.0	3.4–4.5
Retired	998	7.7	6.0–9.4	999	5.2	3.8–6.6
Homemaker/unemployed	469	7.7	5.3–10.1	445	3.4	1.7–5.1
Others	258	8.5	5.1–12.0	207	6.3	2.9–9.6

Between May 21^st^ and July 4^th^, 2014, a total of 306 (4.2%) persons in 7,354 participants reported that they had an episode of influenza like illness (ILI) within 3 months. Similarly, no significant differences were observed in the occurrence of ILI by age, gender, education, and status of marriage and employment.

### Healthcare seeking behaviors

About 61.9% of self-defined ILI cases consulted a physician, usually a general practitioner. The consultation rate during epidemic period is higher than that during non-epidemic period (67.9% vs. 52.3%, *p* < 0.001). Persons older than 60 were more likely to consult a primary care physician. The consultation rate of elder in primary or community healthcare center is more than two times of that in general hospital (levels 2, 3) (47.7% vs. 20.5%, *p* < 0.001).

Table 2 showed the consultation rates in general hospitals (level 2 and 3) of self-defined ILIs during two phases of influenza season. Healthcare usage changed significantly during this influenza season. The proportion of healthcare usage during epidemic period is higher than that was during non-epidemic period (27.1% vs. 19.0%, *p* = 0.008). During epidemic period, the consultation rates of two age groups (18-, 60-) in general hospitals were respectively 27.3 (CI 95% = [22.8, 31.7]) and 26.7 (CI 95% = [18.0, 35.5]). No significant differences between two age groups were observed. The consultation rate decreased in non-epidemic period. Similarly, the difference in the consultation rate between male and female was not statistically significant. During non-epidemic period, there were also no significant differences between different ages and genders in the consultation rate of general hospital for ILI.

**Table 2:** Healthcare seeking behavior for ILI during epidemic and non-epidemic period^a^

Characteristics	Consult a physician during epidemic period		Consult a physician during non- epidemic period	
	% of total (n)	95% CI	% of total (n)	95% CI
All respondents	27.1 (490)	23.2–31.1	19.0(306)	14.5–23.4
Age				
18–59	27.2(389)	22.4–32.2	21.2(231)	15.8–27.2
≥ 60	26.7(101)	18.8–36.1	12.0(75)	5.6–21.2
Sex				
Male	26.5(223)	21.8–32.0	23.4(140)	14.6–31.3
Female	27.7(267)	22.3–33.6	15.2(166)	10.0–20.6
Level of education				
Less than high school	16.0(181)	11.0–21.3	11.4(105)	6.3–18.8
High school graduate	34.0(141)	26.8–44.0	19.60(92)	10.3–28.5
College school graduate	34.3(167)	25.6–41.4	25.7(109)	17.7–35.5
Marriage status				
Married	27.3(396)	13.8–40.2	17.6(261)	9.5–41.8
Divorced	23.3(30)	21.9–32.6	25.0(16)	12.9–23.0
Unmarried	26.7(60)	10.8–38.7	27.6(29)	6.4–48.4
Employment status				
Employed	23.9(339)	18.5–27.9	20.5(215)	14.2–25.1
Retired	40.0(75)	26.5–50.3	15.4(52)	6.7–26.2
Unemployed/other	29.3(75)	18.6–41.2	15.4(45)	2.5–28.6
Medical Insurance				
No	12.1(58)	3.5–23.6	7.1(50)	2.8–19.9
Insured	29.2(432)	24.2–33.3	20.4(255)	15.0–24.7
Residence				
Urban	32.6(233)	27.5–39.6	18.0(184)	12.7–24.9
Suburb	21.9(256)	17.1–26.9	20.3(122)	12.8–27.5

^a^Consultation rates of self-defined ILI cases were limited in general hospitals (level 2 and 3), excluding primary or community health care center

The relationship between healthcare seeking behavior and socio-demographic factors of responds were examined by using multivariate logistic regression analysis. Results showed that level of education, status of medical insurance were statistically associated with seeking healthcare. Compared with individuals who did not seek healthcare during epidemic period, those who did were more likely to be with high level of education and have health insurance. Respondents who are high school graduates (OR 2.252; 95% CI: 1.450–3.498) and college graduates (OR 2.428; 95% CI: 1.592–3.704) showed increased odds of seeking medical usage, comparing with individuals whose educational levels were lower than high school. Persons who have medical insurance reported a higher consultation rate (OR 2.344; 95% CI: 1.012–5.427) than those who had no medical insurance (Table 3).

**Table 3:** Multivariate logistic regression models of respondents’ health-seeking behaviors

Variables	OR (95% CI)
Level of education	
Less than high school (Ref.)	
High school graduate	2.353 (1.370,4.042)*
College school graduate	2.646 (1.555,4.502)*
Status of insurance	
No (Ref.)	
Insured	2.344 (1.012,5.427)*

*p < 0.05

### Estimates of case numbers

**Fig. 2:**
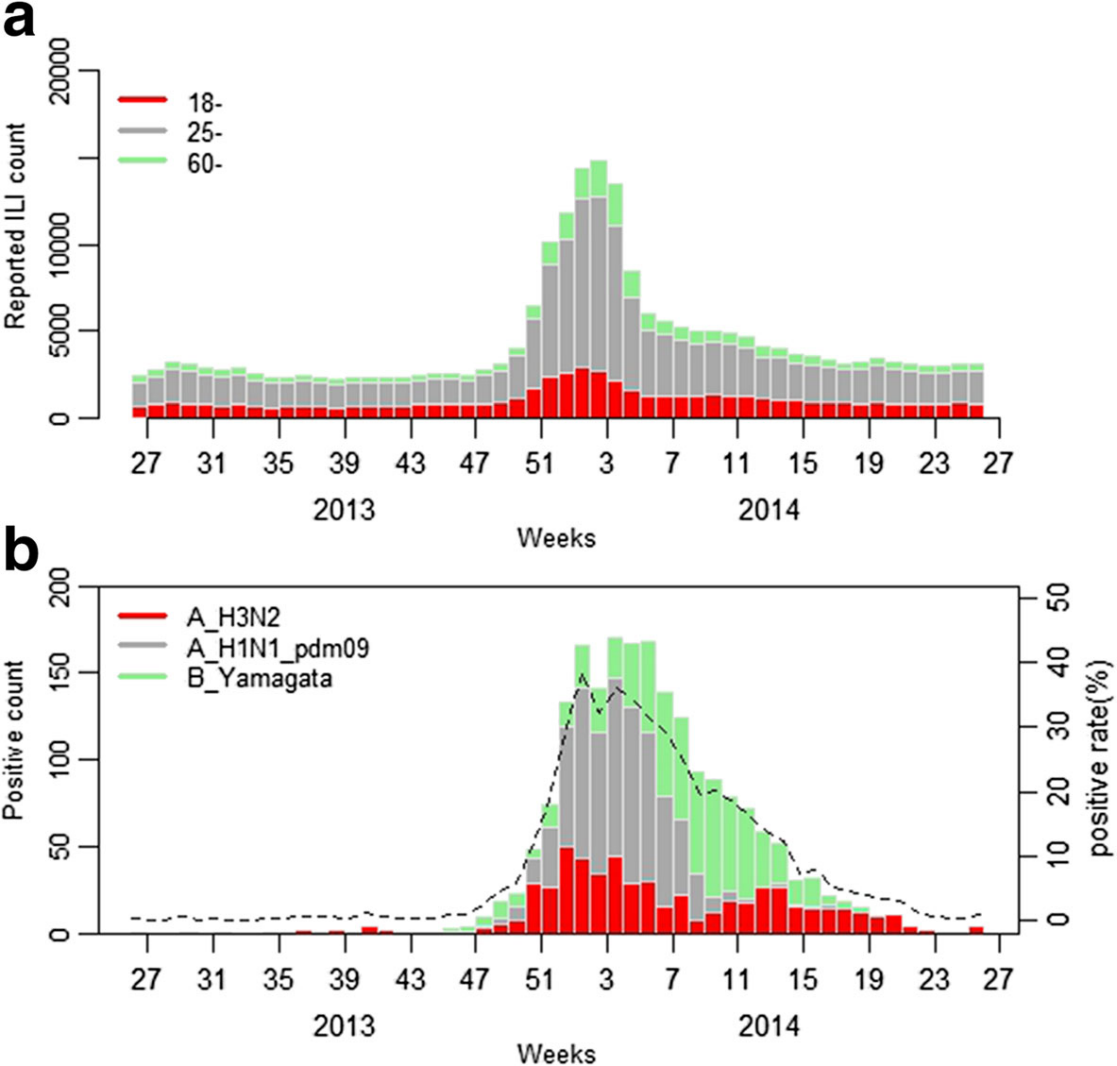
Weekly reported ILI counts by age, positive isolates and total positive rate of influenza virus during 2013–2014 season, Beijing, China* (*During 2013–2014 influenza season, a total of 20,476 pharyngeal swab specimens from the ILIs case-patients (within 3 days of symptom onset from patients who had not received antiviral drugs) were collected by designated staff. 2,334 isolates were positive, with an average positive rate of 11.4%.)

During 2013–2014 influenza season, a total of 258,423 ILI consultations in the adult population were reported in Beijing influenza surveillance system, 61.8% of which were between the age of twenty-five and fifty-nine. 61,790 ILI consultations (23.9%) were aged 18–24 and 36,908 (14.3%) were aged ≥60 years (Fig. 2(a)). Figure 2(b) showed that the weekly positive rate of influenza virus varied significantly with seasons. The overall positive rate increased from July gradually and peaked at week 1, 2014 and then fell to its lowest point at week 25, 2014. Influenza A (H1N1) pdm 09 strains was the predominant influenza virus circulating in the Beijing during this season, accounting 37.8%, followed by B (Yamagata) (34.0%) and A (H3N2) (27.8%).

Based on weekly reported number of ILI consultations and weekly subtype specific positive rate, a total of 54,902 baseline number of influenza cases were estimated, with 52,001 in epidemic period and 2,901 in non-epidemic period. Baseline number of influenza cases by subtype and age was shown in Table 4. Using the baseline number of influenza case, the consultation rate of 23.2–31.1% during epidemic period and 14.5–23.4% during non-epidemic period, and the proportion of ILI among influenza cases of 40.0–70.0%, we estimated that there were 379,767 (90% CI = [281,934, 526,565]) confirmed cases of influenza amongst adults in Beijing during 2013–2014 influenza season (Table 5), with an incidence rate of 2.0%. Influenza A(H1N1) pdm 09 showed a higher incidence rate, comparing with A(H3N2) and B. No significant difference in the incidence rate by age was observed. However, there were statistically significant difference between epidemic period and non-epidemic period. The incidence rate during epidemic period was almost ten times of that in non-epidemic period.

**Table 4:** The baseline number of influenza case during epidemic and non-epidemic periods

Characteristics	Epidemic period	Non-epidemic period
Subtypes		
A(H1N1) pdm 09	25,098	118
A(H3N2)	13,060	1,781
B	13,714	1,002
Age groups		
18–59	44,409	423
≥ 60	7,592	2,478
Total	52,001	2,901

**Table 5:** Estimated numbers of influenza cases and incidence rates, Beijing, China, during 2013–2014 influenza season

Characteristics	Estimated no. influenza cases, median (90% CI)	Estimated rate, %, median (90% CI)
Subtypes		
A(H1N1) pdm 09	170,010 (127,381–234,895)	0.9 (0.7–1.2)
A(H3N2)	105,483 (77,957–145,661)	0.6 (0.4–0.8)
B	103,180 (76,483–139,276)	0.5 (0.4–0.7)
Age groups		
18–59	324,581(240,140–451,216)	2.0 (1.5–2.8)
≥ 60	58,594 (39,416–97,984)	2.2 (1.5–3.6)
Periods		
Epidemic	350,872 (261,419–485,782)	1.9 (1.4–2.6)
Non-epidemic	28,896 (20,515–40,783)	0.2 (0.1–0.2)
Total	379,767 (281,934–526,565)	2.0 (1.5–2.8)

## Discussion

To our knowledge, this is the first time a survey of healthcare seeking behavior has been conducted to estimate the true burden of influenza cases in Beijing, China. In this study, a total of 379,767 episodes of influenza were estimated among adults in Beijing during 2013–2014 influenza season, with an incidence rate of 2.0%. This rate is lower than the previously estimated annual global attack rate at 5–10% in adults [1, 13]. Since there is a lack of published literature about the estimated attack rate of influenza in other regions of China, it is not easy to compare the estimated incidence rate in Beijing with other regions of China. The incidence rate in epidemic period was about ten times of that in non-epidemic period, which is critical for policy makers to effectively decide on strategies regarding influenza (e.g., stockpiling and resource allocation, planning of additional hospital bed capacity, or implementation of vaccination programs).

In addition, we observed that people older than 60 had a higher proportion of healthcare service usage. They were more likely to visit a primary healthcare center or community healthcare center, comparing with visiting a general hospital. Although the specialized service provided by primary or community healthcare centers was not as high as that in secondary or tertiary hospitals, those healthcare centers had their own advantages. Since they were located in communities, the medical service provided is much more accessible and acceptable. In contrast, the proportion of self-defined influenza cases that consulted a physician in general hospital is higher among people aged between 18 and 59. Moreover, we found that individuals who are with higher education level and have medical insurance were more likely to seek healthcare service in general hospital. Lack of insurance and health education reduced healthcare seeking significantly in this study. These findings will be helpful to guide response to and plan for the future pandemic. Additionally, the healthcare-seeking behavior of ILI cases varied during the course of influenza season. Before the epidemic of influenza, consultation rate was lower. However, at the start of the epidemic, there were extensive health education campaigns and high media coverage, which might increase the medical service usage. Consultation rate in epidemic period was estimated higher than that in non-epidemic period. Given this finding, it is difficult to generalize the consultation rate during 2013–2014 influenza seasons to other seasons to estimate the actual annual magnitude of influenza in Beijing. This finding indicated that continuous surveillance of health seeking behavior was essential for better estimates of the magnitude of influenza cases.

The surveillance system for influenza-like illness (ILI) and laboratory confirmed influenza introduced in Beijing provided timely and accurate surveillance information that was consistent with data obtained from virologic surveillance for influenza. The system enabled us to detect the variation in influenza virus activity and the epidemic trend of influenza in time, which facilitated this system to have the potential to provide baseline case number to estimate the actual annual magnitude of influenza [8].

Our study has several potential limitations. In our study, cases were determined by participants themselves according to the definition of ILI, without biological or even practitioner’s confirmation. The definition of self-defined influenza cases was so broad that other diseases with similar symptoms but caused by other pathogens might have included ILI cases. In theory, the estimates of influenza cases should be based on the proportion of probable influenza cases (diagnosed by clinical physicians) seeking healthcare. However, it is not feasible for participants to differentiate influenza from the similar symptoms of other disease. We assumed that amongst self-defined influenza cases, no matter whether they had ever sought healthcare or not, the ratio of probable influenza cases and cases of other diseases was the same. The consultation rate of ILI might be consequently considered as be equal to the consultation rate of probable influenza cases with ILI symptom based on this assumption. We believed, however, that the impact of this assumption is likely to be limited, since our results is consistent with the proportion observed in the Netherlands (25%) and in Portugal (45%) during a seasonal influenza due to A (H3N2) virus [5]. On the other hand, potential recall bias may exist in this study which is common to other retrospective studies. Although we used a two-week recall period during epidemic period to limit recall bias, we used a longer recall period during non-epidemic period due to a lack of ILI case. This might cause recall bias in this study. In addition, participants in the first survey may be included in the second survey. Although this possibility is small, we think this may introduce potential bias in this study.

## Conclusions

Our results showed that the surveillance system for ILI and virological data has the potential to provide baseline case number to estimate the actual annual magnitude of influenza. Given the changes in healthcare seeking behavior over time, sentinel surveillance on healthcare seeking behavior are required to be established for better estimate of the true case number of influenza.

## Abbreviations

CI: Confidential interval
ILI: Influenza-like illness
